# A Framework for Lung and Colon Cancer Diagnosis via Lightweight Deep Learning Models and Transformation Methods

**DOI:** 10.3390/diagnostics12122926

**Published:** 2022-11-23

**Authors:** Omneya Attallah, Muhammet Fatih Aslan, Kadir Sabanci

**Affiliations:** 1 Department of Electronics and Communications Engineering, College of Engineering and Technology, Arab Academy for Science, Technology and Maritime Transport, Alexandria 1029, Egypt; 2 Department of Electrical and Electronics Engineering, Karamanoglu Mehmetbey University, 70100 Karaman, Turkey

**Keywords:** CNN, deep learning, DWT, FHWT, lung and colon cancer diagnosis, PCA

## Abstract

Among the leading causes of mortality and morbidity in people are lung and colon cancers. They may develop concurrently in organs and negatively impact human life. If cancer is not diagnosed in its early stages, there is a great likelihood that it will spread to the two organs. The histopathological detection of such malignancies is one of the most crucial components of effective treatment. Although the process is lengthy and complex, deep learning (DL) techniques have made it feasible to complete it more quickly and accurately, enabling researchers to study a lot more patients in a short time period and for a lot less cost. Earlier studies relied on DL models that require great computational ability and resources. Most of them depended on individual DL models to extract features of high dimension or to perform diagnoses. However, in this study, a framework based on multiple lightweight DL models is proposed for the early detection of lung and colon cancers. The framework utilizes several transformation methods that perform feature reduction and provide a better representation of the data. In this context, histopathology scans are fed into the ShuffleNet, MobileNet, and SqueezeNet models. The number of deep features acquired from these models is subsequently reduced using principal component analysis (PCA) and fast Walsh–Hadamard transform (FHWT) techniques. Following that, discrete wavelet transform (DWT) is used to fuse the FWHT’s reduced features obtained from the three DL models. Additionally, the three DL models’ PCA features are concatenated. Finally, the diminished features as a result of PCA and FHWT-DWT reduction and fusion processes are fed to four distinct machine learning algorithms, reaching the highest accuracy of 99.6%. The results obtained using the proposed framework based on lightweight DL models show that it can distinguish lung and colon cancer variants with a lower number of features and less computational complexity compared to existing methods. They also prove that utilizing transformation methods to reduce features can offer a superior interpretation of the data, thus improving the diagnosis procedure.

## 1. Introduction

Cancer occurs as a result of the uncontrolled proliferation of abnormal cells in the body’s organs or tissues. Cancer cells can occur in different organs or tissues of the body. According to the estimates made by the World Health Organization (WHO) for 2019, the primary or secondary cause of death before age 70 is cancer for 112 countries [1,2]. Furthermore, based on a report published by the International Agency for Research on Cancer (IARC) [3] in 2020, cancer is the primary or secondary cause of death in 134 countries. Cancer has more than 200 types [4]. Lung and colon cancers are anticipated to rank in the top three of the most prevalent cancer types in 2020, according to a statistical analysis done in America. The research also indicated that among all cancer diagnoses in America in 2020, patients with lung and colon malignancies will experience the highest rates of death [5]. Lung and colon cancer rates are 11.4% and 18.0%, respectively, according to GLOBOCAN 2020 statistics [1]. Moreover, the WHO stated that around 4 million people globally developed colon or lung cancer in 2020. About 2.7 million people died from these cancers [6]. These statistical results prove that lung and colon cancer are common and deadly diseases worldwide.

Lung cancer might occur at the same time as colon cancer, as based on [7], about 17% of cases of these two cancers occur simultaneously. In addition, there is a considerable risk of cancer cells spreading between the two organs in the absence of an early diagnosis. Smoking is known to have a detrimental impact on the development of lung cancer, and it is believed that an unaware diet contributes to the development of colon cancer. In other words, the negative effect of lung cancer on the intestines can trigger colon cancer. Therefore, lung cancer can be seen as the second cancer disease in patients with colon cancer [7]. That is, a patient can get both lung and colon cancer at the same time. For this reason, it is vital to investigate both types of cancer in patients together and to diagnose them early [8].

Symptoms that can provide an early diagnosis of cancer are not direct markers of cancer. The most common symptoms such as fatigue, cough, muscle pain, etc., occur along with different types of diseases. The biggest tool that shows the presence of cancer is medical imaging devices [9]. Radiographic imaging methods such as mammography, histopathological imaging, computed tomography (CT), positron emission tomography (PET), magnetic resonance imaging (MRI), and ultrasound are frequently used for cancer detection [9,10]. Among them, histopathology images containing phenotypic information are indispensable for the diagnosis and evaluation of cancer diseases in clinics [10,11]. Manual analysis of such medical images by experts is a delicate and difficult task. It is therefore time consuming and requires a strong focus [10,12]. Moreover, the detection of cases is much more difficult in the case of early diagnosis because at the beginning of the disease, the symptoms are very vague and difficult to diagnose. When symptoms become evident, it is too late for early treatment [9]. Thanks to the advancements achieved in the field of artificial intelligence (AI), today, AI-based medical image analysis methods have assumed the role of a decision support mechanism due to both early diagnosis and support to doctors [13,14].

The automatic diagnosis methods are based on AI technology such as machine learning and deep learning. In this way, data analysis tasks based on expert knowledge have subsequently evolved into expert-independent and fully automatic diagnostic systems [15]. Several conventional machine-learning methods have been utilized to solve medical problems [16,17,18,19,20,21,22] and health-related applications [23,24]. However, these methods primarily require feature selection and feature extraction steps, and they suffer from the disadvantages of not using the appropriate feature extraction method and loss of information during feature extraction. On the other hand, deep learning (DL) has become popular in medical diagnostic applications due to both eliminating these disadvantages and strong discrimination ability [25]. Medical data are usually radiographic image data, and thus the convolutional neural network (CNN) is the well-known DL architecture commonly used to analyze medical images [26]. Recently, both pre-designed and pre-trained CNN models are frequently preferred due to their convenience and high performance. Examples of these models are AlexNet [27], SqueezeNet [28], ResNet [29], ShuffleNet [30], VGGNet [31], MobileNet [32], and GoogleNet [33]. These networks have been extensively used in the medical domain to analyze medical images and diagnose several types of diseases such as heart abnormalities [34], lung anomalies [35,36,37,38,39], brain disorders [40,41,42], breast cancer [43,44,45], genetic facial diseases [46], and eye diseases [47].

CNN models extract high-level features from raw data through their deep layers. In this way, complex and difficult data can be interpreted successfully thanks to CNNs. This success of CNN models is actually due to the complexity and depth of its architecture. As the complexity of the model increases, the number of parameters in the model also increases [48]. Modern CNN models require millions of parameter updates during the training phase. However, the large number of parameters may negatively affect the generalization ability of the network and cause overfitting [49]. Reducing the number of features and utilizing lightweight DL models are solutions to prevent overfitting that might occur due to the complexity of the model [50]. In general, the number of features and parameters of pre-trained CNN models is high, which may cause overfitting and adversely affect classification and/or regression performance. Various feature reduction methods can be used to avoid overfitting; thus, unnecessary and/or redundant features are removed [51]. The most well-known size reduction algorithm is principal component analysis (PCA). Moreover, transformation methods such as fast Walsh–Hadamard transform (FHWT) and discrete wavelet transform (DWT) that analyze data and extract useful representations of the data can reduce their dimension. Thanks to feature reduction and transformation methods, features that do not contribute to network training are reduced, and solutions with less computational complexity are provided [52]. Although accuracy comes to the fore in AI-based medical diagnostic applications, the reliability and computational complexity of the system should also be considered. A network with less computation is preferable to a more complex network with similar accuracy. 

This study presents a framework based on multiple lightweight DL models and transformation methods for the early detection of lung and colon cancer, which has a striking number of cases and deaths in both men and women worldwide. Experimental studies are performed on the five-class LC25000 dataset containing histopathological images for colon cancer and lung cancer. Unlike previous work, both accuracy and less computational complexity are adopted in principle. In this context, after preprocessing involving resizing and data augmentation, histological images are fed into ShuffleNet, MobileNet, and SqueezeNet models, which are lightweight DL architectures. PCA and FHWT methods are used to reduce the size and complexity of deep features obtained from these CNN models. Afterwards, reduced features of the FWHT obtained from the three DL models are fused using DWT. Additionally, PCA features attained using the three DL models are concatenated. As a result of PCA and FHWT reduction and fusion steps, the reduced features for each model are given as inputs to four different machine learning algorithms (LDA, QDA, SVM, and ensemble subspace discriminate (ESD)), both individually and combined. The classification accuracies obtained from machine learning algorithms show that the proposed methodology can distinguish lung and colon cancer variants with less computational complexity and high accuracy.

The key novelty and contributions of the introduced framework are:

- The proposed framework depends on lightweight deep learning models, including ShuffleNet, MobileNet, and SqueezeNet, in contrast to many of the earlier studies based on heavy deep learning models such as VGG.
- The majority of related studies relies on individual deep learning models for performing the diagnosis; nevertheless, the proposed framework utilizes three deep learning models of various structures.
- Most previous studies used spatial deep features extracted from the last pooling layer of the deep learning models directly to train machine learning classifiers, which may have huge dimensions and thus increase the complexity and duration of the classification process; however, the proposed framework employs two feature reduction approaches to diminish their dimension, including PCA and FHWT.
- The reduced sets of features generated after FHWT provides the spatial–frequency demonstration of the features, not only spatial information such as with previous methods.
- The proposed framework integrates the privileges of distinct architectures of three deep learning models by first merging the reduced features obtained using PCA for the three deep learning models.
- Furthermore, the three reduced sets of features produced after FHWT for ShuffleNet, MobileNet, and SqueezeNet are merged using DWT, which offers the time–frequency representations of the features of lung and colon cancer, resulting in spatial–time–frequency representations, which usually improves diagnostic performance and further lowers the features dimension.

## 2. Literature Survey

The proposed method in this study is analyzed on the LC25000 lung and colon cancer histopathological image dataset published in 2020. Because lung and colon cancer cases are frequent, the dataset is new and contains a suitable number of images for deep learning, and many researchers have recently implemented deep learning-based applications on this dataset. Some of these are explained below.

Masud, Sikder, Nahid, Bairagi, and AlZain [9] presented a classification system for five types of lung and colon tissues by employing deep learning on histopathological images. First, pathological sample images were subjected to image sharpening. Then, the features were extracted from the images with a 2D-Fourier transform and 2D wavelet transform. These features were used to train a manually tuned CNN model. It was reported that the accuracy performance of this model was 96.33%. Alternatively, Kumar, Sharma, Singh, Madan, and Mehandia [8] compared two classification methods for lung and colon cancer. In the first presented method, texture-, color-, and shape-based features were extracted from histopathological images. These attributes were used for classification with various machine learning methods such as random forest, multilayer perceptron, support vector machine (SVM) with radial basis function, and gradient boosting. In the second method, transfer learning (TL) was used for feature extraction. For this purpose, seven pre-trained CNN models were used for feature extraction. The extracted deep features were applied to the same machine learning methods for classification. It was reported that the random forest algorithm had the best performance with attributes obtained by the TL method from DenseNet-121. The performance of this couple was 98.60% in terms of accuracy. Talukder et al. [53] presented a hybrid ensemble attribute-obtaining method for the identification of lung and colon cancer types. The deep feature extraction method and ensemble learning for image filtering were integrated. It was reported that the proposed hybrid model detected cancer possibility with a rate of 99.05%, whereas Mangal et al. [54] proposed a CNN-based diagnosis system. A shallow neural network architecture was employed for the classification of samples as squamous cell carcinomas, adenocarcinomas, and benign. For lung and colon samples, separate training was performed. It was reported that the success rates of the presented models were 97% and 96% for lung and colon, respectively. Similarly, Hatuwal and Thapa [55] presented a CNN-based histopathological image classification method for cancer diagnosis. A custom-shaped neural network was created and trained. It was reported that the training and validation accuracies were 96.11% and 97.20%, respectively. In contrast, Ali and Ali [56] proposed a multi-input capsule network model for the diagnosis of lung and colon cancer. CLB (convolutional layers block) and SCLB (separable convolutional layers block)) convolutional layers were used in this model. By using the dual input technique, the model learned the features more efficiently. Their proposed model achieved 99.58% accuracy for lung and colon abnormalities based on histopathological images. On the other hand, Hasan et al. [57] used the deep CNN model for detecting and classifying colon cancer using colon images of the LC25000 dataset. In this colon data, there were 10,000 histopathological images of colon samples. The operations performed on the images split the data as 80% training, 10% validation, and 10% testing, data augmentation in the separated groups, normalization, and feature extraction with PCA. A ten-layered custom CNN model was constructed and trained. The images are classified as adenocarcinoma or benign. Success rates obtained from different pre-trained CNN models with transfer learning (TL) and classification operations and the results of the specified method were compared. Success rates obtained from different pre-trained CNN models with TL and classification operations and the results of the specified method were compared. In addition, Bukhari et al. [58] used three CNN models, ResNet18, ResNet30, and ResNet50, to classify digital images of colon tissue. The publicly available LC25000 and (colorectal adenocarcinoma gland) CRAG datasets were used in this study. The colon images of the LC25000 dataset were used to train the models and for validation phases. The CRAG dataset was used for the training and testing phases. The highest classification accuracy of 93.13% was achieved with the Resnet50 model.

When previous studies are examined in general, deep features extracted with different CNN models are classified by either end-to-end DL or TL and machine learning techniques. Furthermore, these studies depended on DL models, which necessitate a high level of computational ability and resources. In addition, the majority of them relied on individual DL models to extract high-dimension features or perform diagnosis. However, our study, unlike previous studies, uses multiple lightweight CNN models and adopts feature transformation methods that perform reduction and provide a better representation of the data approaches for reliable and robust diagnosis.

## 3. Materials and Methods

### 3.1. LC25000 Dataset

**Figure 1:**
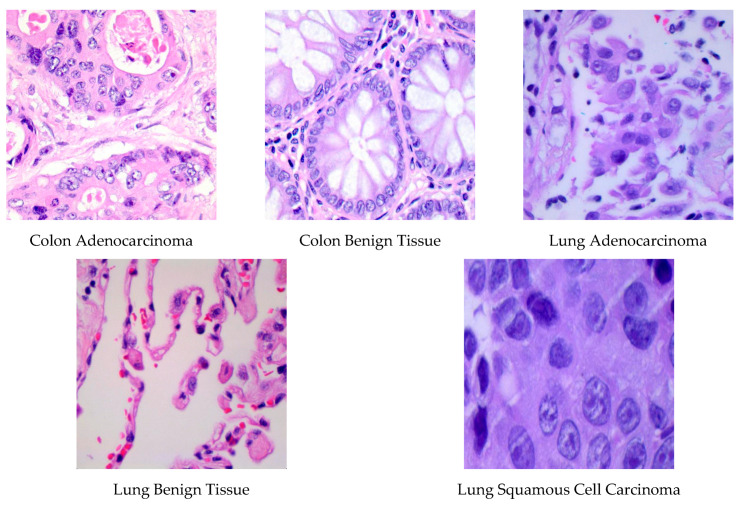
Lung and colon image samples of the dataset.

The LC25000 dataset, created by Borkowski et al. [59] in 2019, contains cancerous samples of lung and colon tissues. This dataset has been approved by the Health Insurance Portability and Accountability Act (HIPAA). It contains lung and colon tissue images. Lung images are classified into three classes: lung benign tissue, lung adenocarcinoma, and lung squamous cell carcinoma. Colon sample images consist of colon benign tissue and colon adenocarcinoma classes. Example for each class of lung and colon images are shown in Figure 1. In the dataset, which consists of five classes in total, there are 5000 samples in each class. As a result, this dataset contains a total of 15,000 images from three-class lung tissue and 10,000 images from two-class colon tissue.

### 3.2. Feature Transformation Approaches

#### 3.2.1. Discrete Wavelet Transform 

Wavelet transform (WT) is a powerful method for the time–frequency analysis of a signal. Although Fourier transform (FT) is also used for frequency analysis, time information is lost after FT. Therefore, it cannot be known which frequency occurs in which time period. Short-time FT (STFT), which is a modification of FT, also includes time resolution by applying FTs at a certain interval [60]. However, fixed-width windows cannot correctly analyze non-stationary signals. WT performs a calculation with variable window sizes to provide more precise time–frequency resolution. In this way, the analysis of non-stationary signals, whose frequency changes over time, is performed in a powerful way [61]. A mother wavelet is determined for WT, and then the wavelet function is scaled and translated. After scaling and translation, the wavelet function is multiplied by the signal and summed over the time domain. These processes continue throughout the signal. In continuous wavelet transform (CWT), the transformation is performed by scaling and translating the wavelet function over the entire time interval. Because this requires excessive computation, the scaling and translation values are usually set as discrete values (discrete wavelet transform (DWT)). When DWT is applied to the image, operations are performed in two dimensions. As a result, four matrices are formed. Three of them (horizontal, vertical, diagonal) represent the detail coefficient, while one represents the approximate coefficient. The detail coefficients are the result of the high-pass filter, and the approximate coefficients are the result of the low-pass filter. Therefore, the edges are more prominent in the detail coefficients. Detail coefficients represent vertical, horizontal, and diagonal coefficients [37].

#### 3.2.2. Principal Component Analysis

Features extracted from a dataset do not contain equal information for classification or regression. Some features are more related to output. Moreover, in artificial intelligence-based applications, high-dimensional features increase the memory requirement, computational cost, and the possibility of overfitting. For this reason, using feature selection or feature reduction steps before classification instead of using direct CNN models with many features can increase the efficiency of the network. To create a feature vector that better represents the output data, either useful features are selected, or different transformations are applied to existing features [62,63]. The most well-known method for size reduction is PCA.

PCA reduces the size of high-dimensional input features by calculating the correlation between them. However, during size reduction, it statistically analyzes the data and generates high variance features at the output. It uses orthogonal transformation techniques to achieve this. In general terms, the features obtained as a result of PCA are formed as a result of projection from multidimensional space to a lower dimensional space. As a result of PCA, new features are both less dimensional and more distinguishable. The features that result from PCA are called principal components. There is no correlation between the principal components, that is, the principal components are perpendicular to each other in the new space created by PCA [63,64].

#### 3.2.3. Fast Walsh–Hadamard Transform

Walsh–Hadamard transform (WHT), which is one of the linear image transformations, is a square wave transform, which is frequently used in signal and image processing, orthogonal and non-sinusoidal. After conversion, a signal is decomposed into a series of functions. These functions are known as Walsh functions. Walsh functions only take two values, +1 and −1. Equation (1) shown below is used to apply the WHT to an array f(x) with n samples [65].

(1) $$F\left(X\right)=\frac{1}{n}\times \left[H_{n}\right]\times f\left(x\right)$$

Here, F(X) represents the WHT applied to the f(x) signal. $\left[H_{n}\right]$ is the n-dimensional Hadamard matrix. The Hadamard matrix is a square matrix of size n×n, and for n = 2, it is as follows:

(2) $$H_{2}=\left(\begin{array}{cc} 1 & 1\\ 1 & -1 \end{array}\right)$$

WHT features simple and fast conversion and is therefore useful for real-time signal and image processing applications. It has the least computational cost among discrete orthogonal transformations [66,67]. With fast Walsh–Hadamard transform (FHWT), WHT is calculated more quickly and efficiently.

### 3.3. Proposed Framework

**Figure 2:**
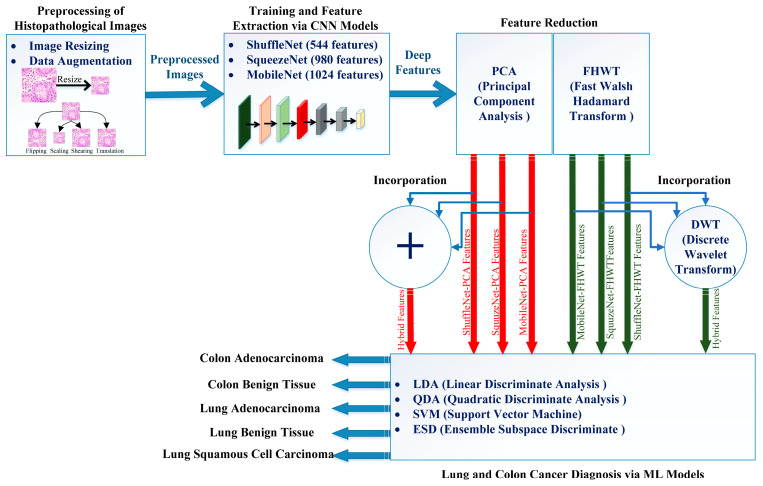
Stages of the proposed framework.

The proposed framework is made up of four stages, which are preprocessing of histopathological images, deep learning models training and feature extraction, feature reduction and incorporation, and finally classification. Firstly, the histopathological image sizes are altered, and then these images are augmented. Next, three pre-trained lightweight deep learning models including ShuffleNet, SqueezeNet, and MobileNet are constructed, and then deep features are extracted. Then, deep features extracted using each deep learning model are reduced using two feature reduction methods. Furthermore, for each reduction approach, the reduced deep features obtained from each deep learning model are incorporated. Finally, these incorporated features are used to train a number of machine learning classifiers. The four stages of the proposed framework are summarized in Figure 2.

#### 3.3.1. Preprocessing of Histopathological Images

The input layer of each CNN accepts an input image of a specific size in order to start the training procedure Thus, the histopathological images dimension of lung and colon cancer are initially altered to be similar to the input layer size of the three deep learning models. The input layer size for ShuffleNet, and MobileNet is 224 × 224 × 3, while for SqueezeNet it is 227 × 227 × 3. Next, in order to improve the training performance of the deep learning models and avoid overfitting, an augmentation procedure is required. Augmentation simply boosts the amount of training images available in a dataset, which allows the training models to learn more effectively [68]. Thus, several augmentation methods are utilized in this paper involving the following: scaling in x and y preferences in the range of [0.5, 2], flipping in both x and y orientations, translation in both x and y orientations with an angle range [−20, 20], and shearing in both directions (x and y) within the range [−45, 45].

#### 3.3.2. Deep Learning Models Training and Feature Extraction

As mentioned earlier, three pre-trained lightweight convolutional neural networks (CNNs) are used in this stage including ShuffleNet, SqueezeNet, and MobileNet. Pretrained CNN means that the network has been previously trained with a large dataset. Transfer learning [69] allows pre-trained CNNs to realize representations from a large dataset of images, such as those in ImageNet, and then apply that knowledge to a related classification problem with smaller datasets, advancing the training process. Therefore, TL is used along with the three pre-trained CNNs. TL is used to modify the number of output layers to five to be equivalent to the number of categories in the dataset. In addition, other CNNs parameters are modified, which will be explained later. Then the three pre-trained CNNs are retrained with the lung–colon cancer dataset. Next, TL is further used to extract deep features from a particular layer of each CNN. In order to produce a more accurate image classification, the CNN first learns the simple patterns of the input image before moving on to learn the important elements of the input image in subsequent, deeper layers [35,36]. Thus, deeper layers are used for feature extraction. The layers used for features extraction are the layers called “node200” for ShuffleNet, “global_average_pooling2d_1” for MobileNet, which are the last pooling layers of these CNNs, whereas for SqueezeNet, the layer “relu_conv10” is utilized for feature extraction, which is a rectified linear unit layer immediately following the tenth convolutional layer. The length of each of the feature vectors extracted is 544, 980, and 1280 for Shuffle, SqueezeNet, and MobileNet, respectively.

#### 3.3.3. Feature Reduction and Incorporation

The dimensions of the feature extracted in the previous steps are large, which could increase the complexity and the duration of the training of machine learning classifiers. Hence, in this stage, two feature reduction approaches are used and compared to lower features dimension and extract a significantly reduced representation of features. These methods include principal component analysis (PCA) and fast Walsh–Hadamard transform (FHWT). Each deep feature set extract from every CNN is analyzed utilizing PCA. Several numbers of principal components are used to study the effect of changing these numbers on diagnostic performance. Similarly, the deep feature sets of the CNNs are analyzed using FWHT, which presents the spectral representations of the input. Different reduced set extracts using FHWT are also employed to examine the influence of varying these numbers on the diagnostic performance. An incorporation step follows the two feature reduction procedures. For PCA, principal components obtained using the three CNNs are concatenated. On the other hand, the reduced feature sets of the three CNNs generated using FWHT are incorporated using DWT, which further diminishes their dimension.

#### 3.3.4. Lung and Colon Cancer Diagnosis

Four machine learning classifiers are used in this stage to diagnose lung and colon cancer and identify the five classes of the lung and colon cancer dataset. These classifiers are LDA, QDA, and SVM with linear kernel function, and ensemble subspace discriminate (ESD). The number of learners and subspace for the ESD classifiers is 30 and 2, respectively, while its learning rate is 0.1. Five-fold-cross validation is employed to evaluate the performance of the proposed CAD. 

## 4. Performance Measures and CNNs’ Parameters Setting

### 4.1. Performance Measures

Several evaluation criteria are used to assess the effectiveness of the proposed pipeline, including precision, accuracy, F1-score, sensitivity, Mathew correlation coefficient (*MCC*), and specificity. The ensuing formulae are used to calculate these metrics (3)–(8). The confusion matrix and receiving operating characteristic (*ROC*) are also computed.

(3) $$Accuracy=\frac{TP+TN}{TN+FP+FN+TP}\;$$

(4) $$Sensitivity=\frac{TP}{TP+FN}\;$$

(5) $$Precision=\frac{TP}{TP+FP}\;$$

(6) $$MCC=\frac{TP\times TN-FP\times FN}{\sqrt{\left(TP+FP\right)\left(TP+FN\right)\left(TN+FP\right)\left(TN+FN\right)}}\;$$

(7) $$F1-Score=\frac{2\times TP}{\left(2\times TP\right)+FP+FN}\;$$

(8) $$Specificity=\frac{TN}{TN+FP}\;$$

The true positive (*TP*) represents the proportion of instances that are perfectly recognized as positive, the false negative (*FN*) denotes the proportion of samples that have been mistakenly categorized as negative, the true negative (*TN*) represents the proportion of instances that are correctly identified as negative, and the false positive (*FP*) resembles the proportion of samples that are erroneously classified as positive.

### 4.2. CNN’s Parameters Setting

The learning rate, frequency of epochs, and minimum batch size were adjusted to 0.0001, 5, and 4, respectively, for retraining the CNNs for end-to-end classification. Furthermore, the validation frequency was set to 130 to calculate the training error per epoch. The stochastic gradient descent with a momentum technique was applied to train the three CNNs. All other hyperparameters remained the same. MATLAB R2020a was employed in implementing the proposed framework.

## 5. Results

### 5.1. Results of PCA

This section examines the performance of reduced features as a result of applying PCA to features extracted from CNN models. These reduced features were individually fed into four different machine learning methods. As a result of applying PCA to deep features, new features are formed according to the number of principal components determined. Therefore, the number of principal components has a direct impact on classification performance. According to the change in the number of principal components, the diagnostic performance of machine learning algorithms also changes. Table 1 shows the variation in the diagnostic performance of different machine learning algorithms as a result of processing the extracted features from SqueezeNet, ShuffleNet, and MobileNet CNN models with PCA with different principal component values. In general, all machine learning algorithms showed outstanding success for lung and colon cancer diagnosis. However, as can be seen from Table 1, the change in PCA values had a significant effect on the accuracy of machine learning methods, because as the PCA value changed, both the value of the features and the number of features changed. As the principal component value was increased from 5 to 30, the diagnostic accuracy of the ShuffleNet-LDA fabric increased by 4.9%. Accuracies for SqueezeNet-LDA and MobileNet-LDA structures increased by 4.2% and 1% with an increasing number of principal components. The accuracy changes for other models (ShuffleNet-QDA, SqueezeNet-SVM, MobileNet-ESD, etc.) are also clearly visible in Table 1.

**Figure 3:**
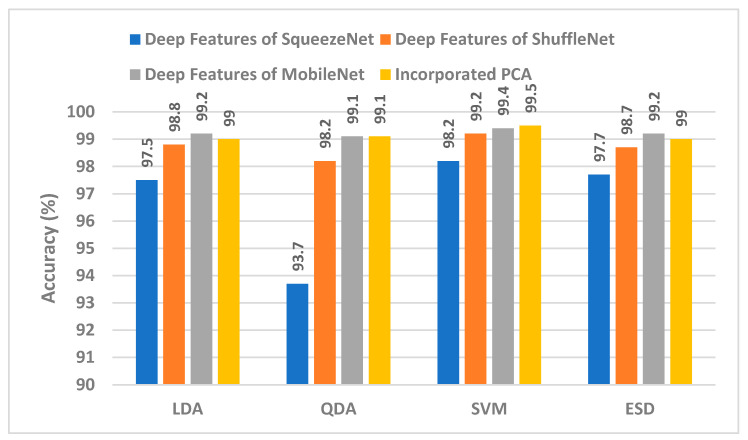
Classification accuracies (%) of deep features versus integrated PCA features of the three CNNs.

**Figure 4:**
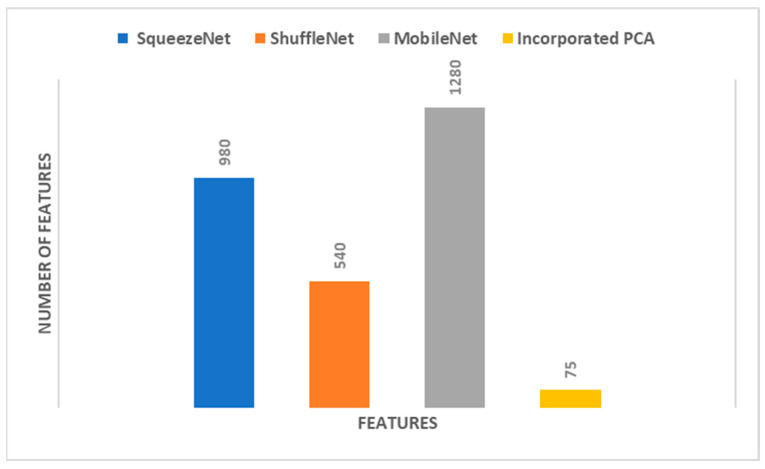
Number of features of each of the deep features extracted from the three CNNs compared to the incorporated reduced features of PCA used to train the four machine learning classifiers.

When Table 1 is examined to compare the success of CNN model features, it is seen that machine learning algorithms classified MobileNet features more accurately. Figure 3 shows the accuracies obtained as a result of the classification of features extracted from all three CNN models by four different machine learning algorithms. The superior performance of MobileNet compared to others can also be seen in Figure 3. Figure 3 also includes the classification accuracies obtained from PCA reduction of the combined features of the three CNN models (incorporated PCA). PCA significantly reduced the combined features of the three CNN models. Figure 4 shows the features extracted from the CNN models and the number of new features obtained by PCA. Accordingly, hybrid features provided very little computational cost compared to CNN models. Note that in Table 1, the highest performance is attained with 15 PCA features for SqueezeNet, 30 PCA features for ShuffleNet, and 30 PCA features for MobileNet. Therefore, when concatenating these PCA features, the total number of incorporated PCA features will be 75, and that is the reason why in Figure 4, the number of PCA features is 75.

**Figure 5:**
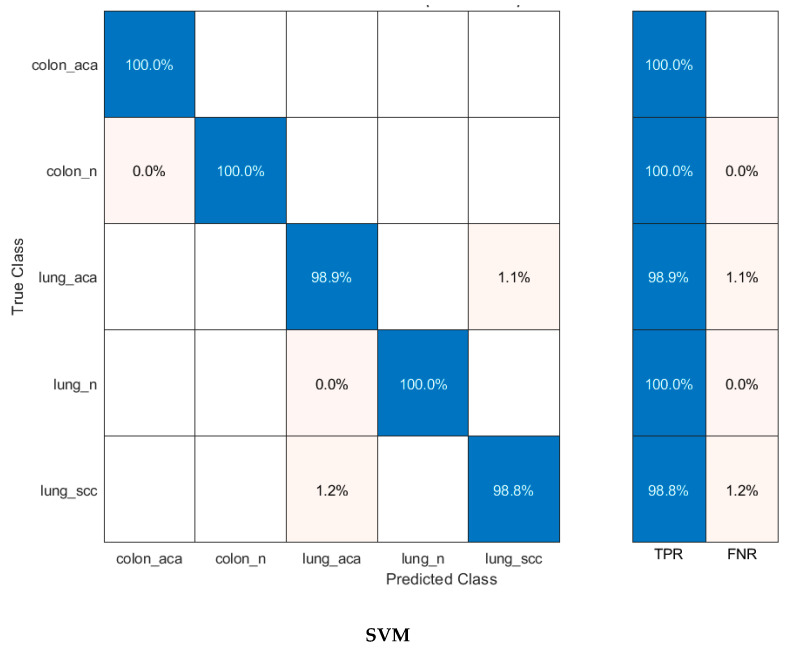

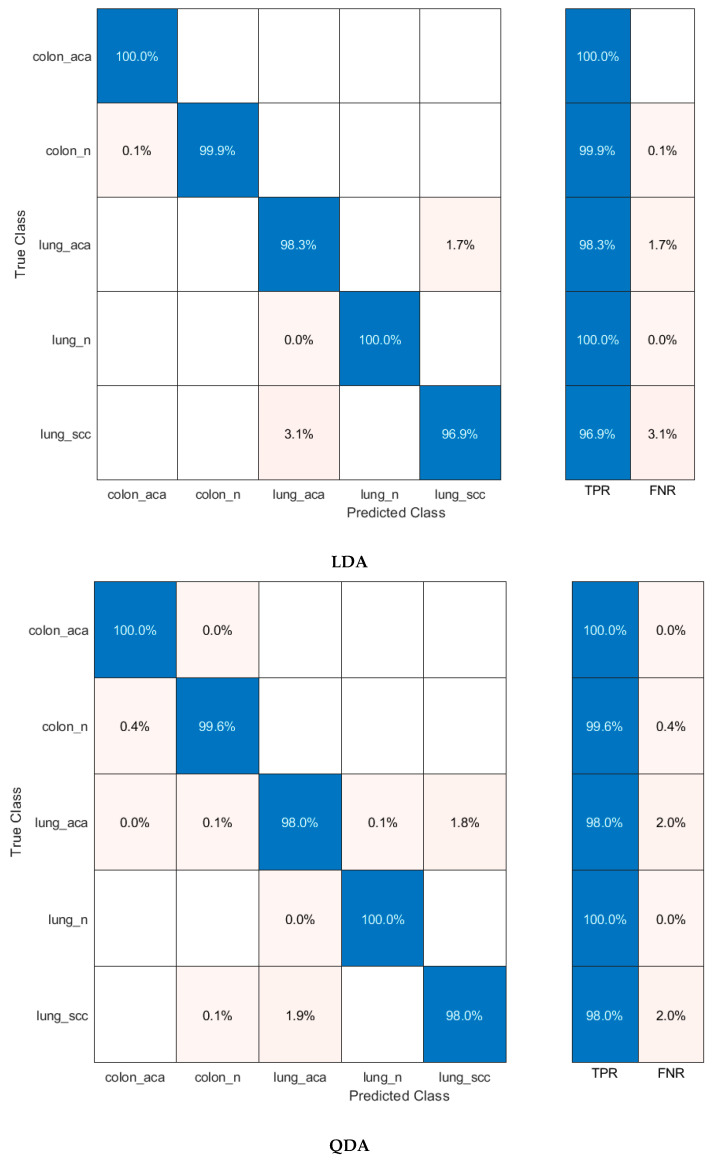

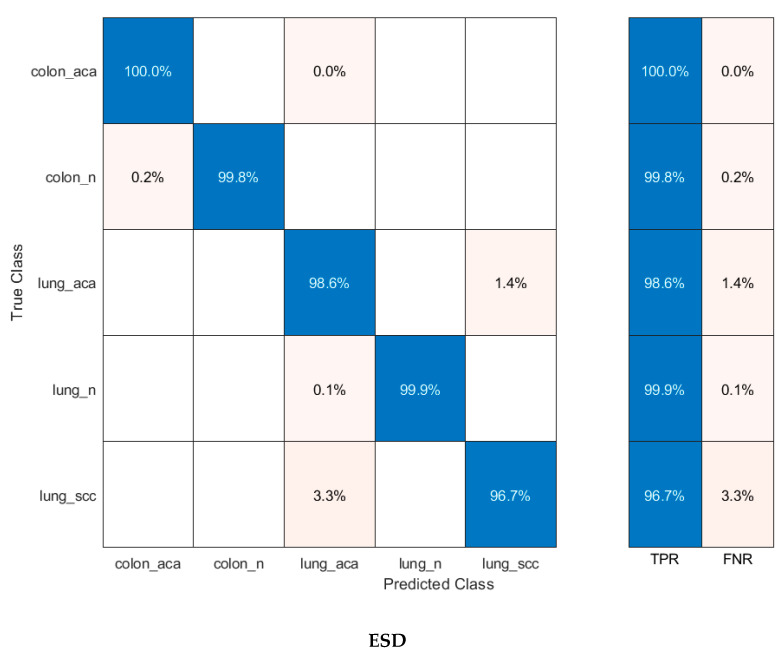
Confusion matrices obtained as a result of classification of PCA-based incorporated CNN features with machine learning algorithms.

**Figure 6:**
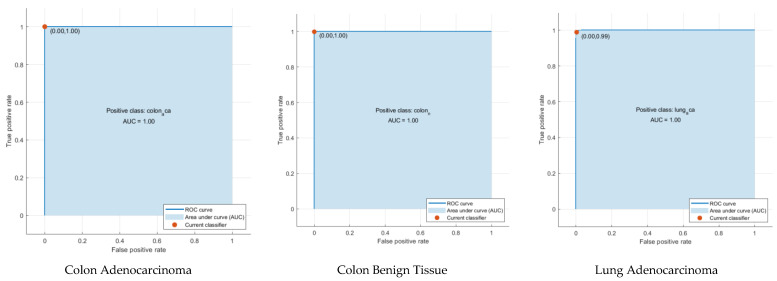

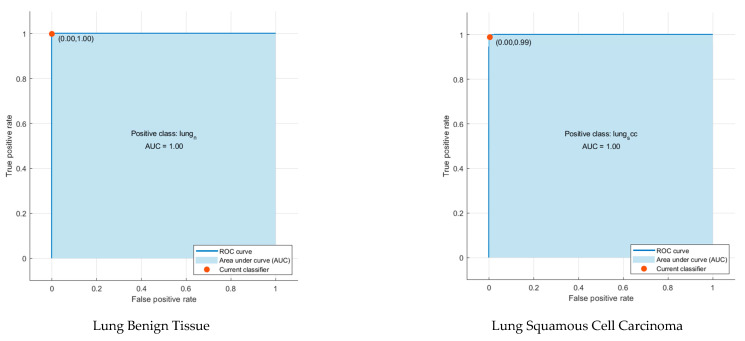
ROC curves of each class as a result of the CNN-PCA-SVM structure.

According to Figure 3, as a result of the classification of hybrid features with machine learning, the highest accuracy was obtained with SVM at 99.5%. Accuracies from other hybrid features were 99%, 99.1%, and 99% for LDA, QDA, and ESD, respectively. Confusion matrices showing the correct and incorrect classification percentages for each class are given in Figure 5. Considering the confusion matrices, all of the colon adenocarcinoma class types were correctly recognized by all machine learning algorithms. In addition, the lung benign tissue class contained 0.1% error in the result of QDA, while it was classified with 100% accuracy by other algorithms. According to the results of the four machine learning algorithms, lung squamous class samples were more difficult to recognize than other classes. Therefore, the error made for this class was higher than for other classes. Machine learning methods generally classify samples belonging to the lung squamous class as lung adenocarcinoma. As can be seen in Figure 5, ESD is the machine learning algorithm that made the most incorrect classifications in the images belonging to the lung squamous class. However, ESD correctly classified 96.7% of test samples belonging to the lung squamous class. Table 2 shows the different performance metrics obtained from the confusion matrices for each machine learning algorithm. When the sensitivity, specificity, precision, F1-score, and MCC metric values were analyzed, the SVM method with linear kernel function produced the most successful results. These results were 0.996, 0.999, 0.996, 0.996, and 0.994, respectively. The most erroneous results were obtained as a result of ESD. Unlike these metric values, receiver operating characteristic (ROC) curves, a popular method to evaluate the accuracy of medical diagnostic systems, were also plotted, as seen in Figure 6 for SVM, which was the most successful machine learning method. This curve was created by plotting the true positive rate against the false positive rate. For successful classification, the area under the curve (AUC) should be close to 1 [70]. As seen in Figure 6, the AUC value was calculated as 1 for all curves. According to these results, the proposed method based on CNN and PCA methods provides a low-cost, unbiased, and highly accurate diagnosis of lung and colon cancer. 

### 5.2. Results of FWHT

This section examines the performance of reduced features as a result of applying FHWT to features extracted from CNN models. FHWT, which is used for feature reduction, has a low computational cost and is preferred in real-time applications because it is a fast conversion tool. In this study, deep features extracted with CNN models were reduced by FHWT. Then these features were classified by machine learning methods. After feature reduction with FHWT, machine learning results differed based on the number of new features. To show the effect of feature reduction on diagnostic performance, different feature size values and corresponding machine learning algorithm accuracy values are shown in Table 3. When the feature sizes and accuracy values were examined, the increase in the number of features decreased the accuracy of the SqueezeNet model. For the ShuffleNet and MobileNet models, the higher the number of features, the higher the accuracy. Accordingly, as the number of features increased, the accuracy of the ShuffleNet-QDA and MobileNet-QDA structures increases by 4.1% and 2.8%, respectively. For SqueezeNet-QDA, increasing the number of features reduced the accuracy by 2.4%.

**Figure 7:**
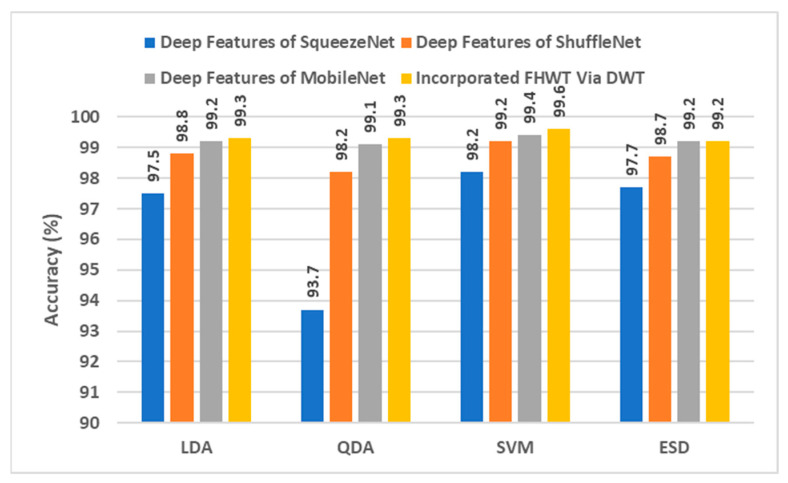
Classification accuracies (%) of deep features versus integrated FWHT features via DWT of the three CNNs.

**Figure 8:**
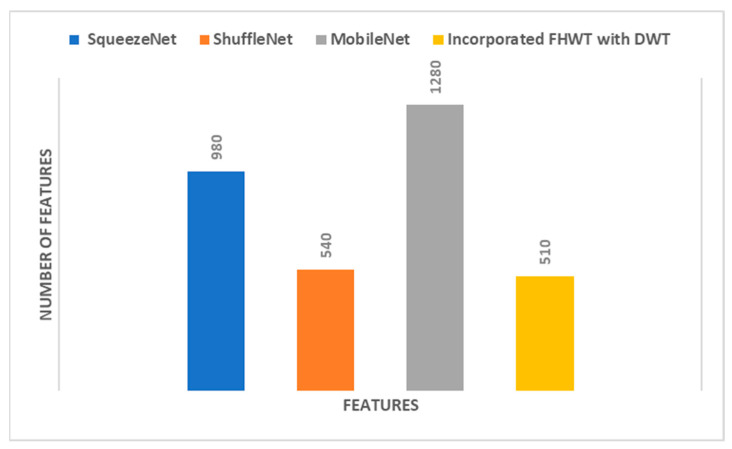
The number of features of each of the deep features extracted from the three CNN compared to the incorporated reduced features of FWHT used to train the four machine learning classifiers.

**Figure 9:**
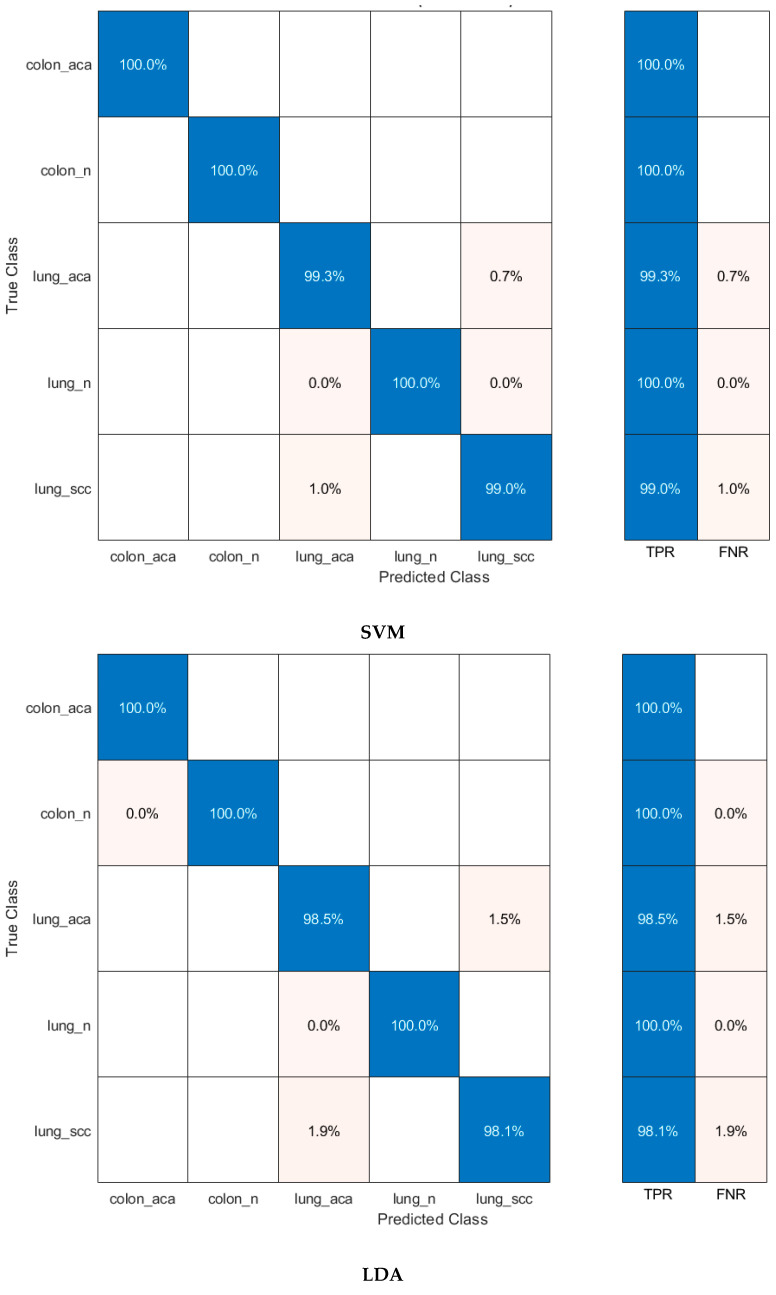

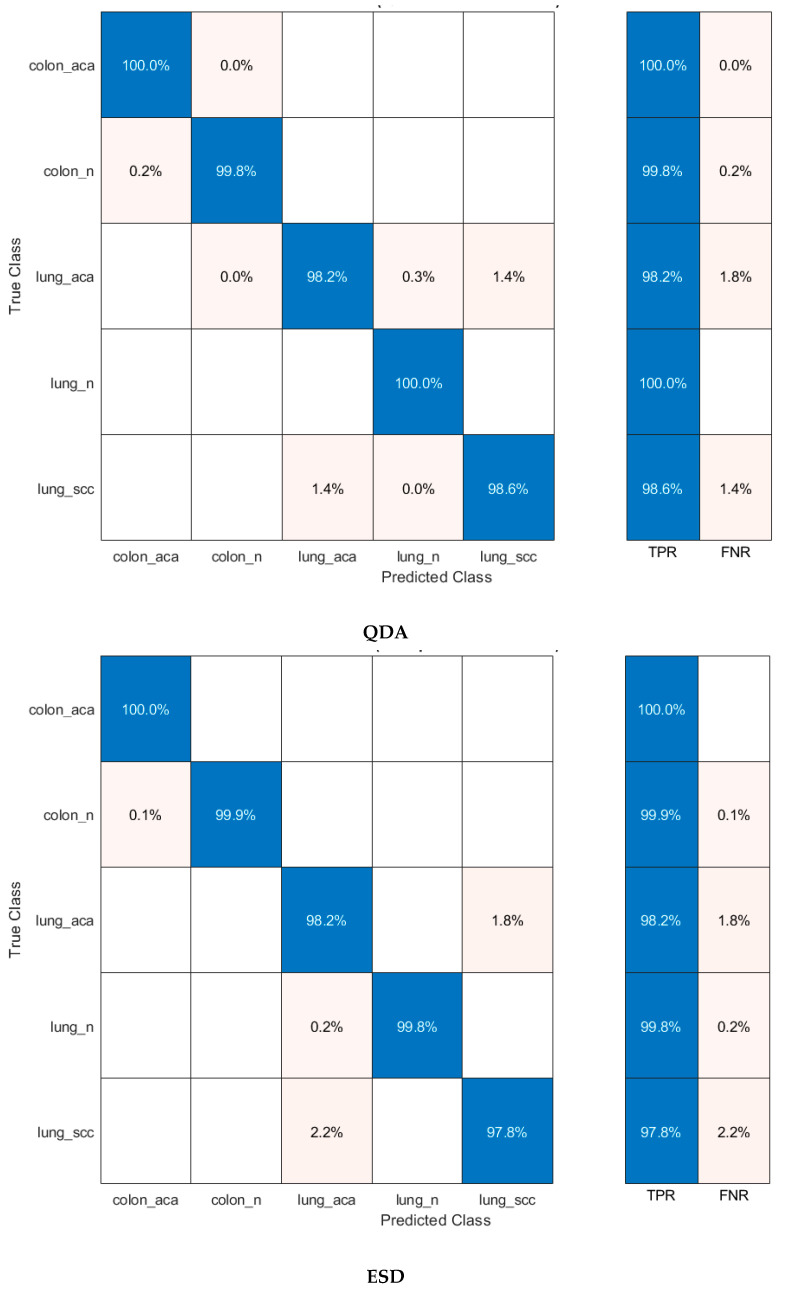
Confusion matrices obtained as a result of the classification of FHWT-based incorporated CNN features using DWT with machine learning algorithms.

**Figure 10:**
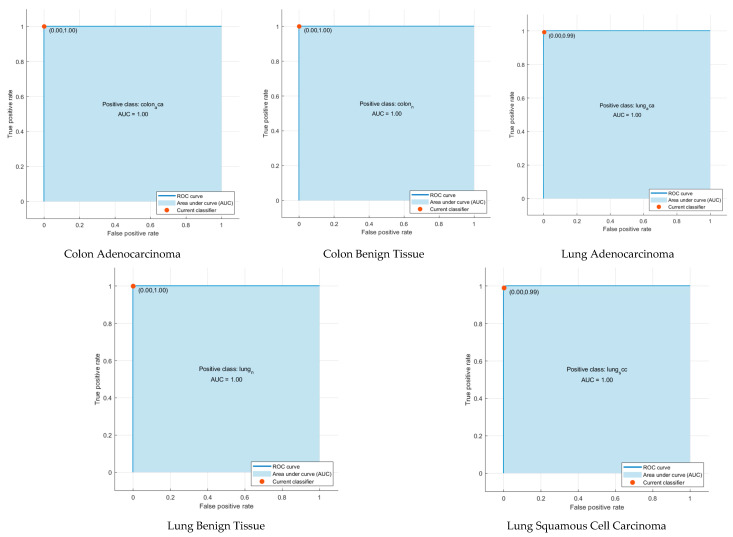
ROC curves of each class as a result of the CNN-FHWT-SVM structure.

When Table 3 is examined to compare the success of CNN models, MobileNet’s superiority over other models is striking, as in the PCA analysis. This can be seen more clearly in Figure 7. Each bar represents a different CNN-machine learning structure so that the success of CNN features and machine learning algorithms can be compared more easily. Figure 7 compares the performance of the deep features of each CNN with the incorporated FHWT features of the three CNNs using DWT. All four machine learning methods classified MobileNet features more accurately. Accordingly, it is understood that MobileNet features represent cancer types more strongly. The most unsuccessful classification was carried out with SqueezeNet features. Figure 7 also includes classification accuracies of hybrid CNN features reduced by FHWT and incorporated with DWT. Accordingly, it is seen that hybrid features created using DWT were better classified by machine learning algorithms. The number of each CNN deep feature and the number of combined features created at the end of the incorporation are shown in Figure 8. This figure shows that the number of FHWT features combined with DWT was much less than other CNN models. Thanks to the feature reduction methods used, more successful results were obtained with fewer features. This also means less computational costs and more reliable results. Accordingly, although hybrid features include all CNN features, they contain fewer features than other models. Furthermore, Figure 7 shows that machine learning algorithms classified hybrid features more accurately than individual features. The most successful method of classifying hybrid features was SVM, and its accuracy value was 99.6%. The accuracies obtained from the hybrid features for LDA, QDA, and ESD were 99.3%, 99.3%, and 99.2%, respectively. The confusion matrices obtained during the classification of hybrid features are also shown in Figure 9. Based on the confusion matrices, colon adenocarcinoma was distinguished with 100% accuracy by all machine learning models. In addition, colon benign tissue and lung benign tissue classes were classified with 100% accuracy by SVM and LDA. Machine learning methods generally made the most inaccurate classification for the lung squamous and lung adenocarcinoma classes. In the misidentifications, some samples belonging to the lung squamous class were classified as lung adenocarcinoma. Similarly, some lung adenocarcinoma samples were assigned to the lung squamous class. The fact that the features in these two classes are similar to each other caused these misclassifications. ESD, which had the lowest average accuracy among machine learning algorithms, classified lung adenocarcinoma and lung squamous classes with 98.2% and 97.8% accuracy, respectively. Table 4 shows the different performance metrics calculated as a result of classification with machine learning algorithms. Similar to accuracy values, while the most successful metric values were provided by SVM, the method with the highest error rate was ESD. Sensitivity, specificity, precision, F1-score, and MCC metric values for SVM were 0.996, 0.999, 0.996, 0.996, and 0.995, respectively. In addition, as in the analysis with PCA, ROC curves were plotted for the most successful machine learning method, SVM, in this section. It is seen in Figure 10 that AUC values are 1 in these curves drawn for each class.

### 5.3. Comparison with the Literature

A comparison was conducted in this section to compare the results of the proposed framework with methods available in the literature. Table 5 demonstrates the results of this comparison. The performance of the proposed framework was competitive with other models employed in the literature. This is because accuracies attained after PCA and FHWT fusion were 99.5% and 99.6%. These accuracies were higher than most of the methods in the literature based on using a single DL model having high computation capacity. Thus, the system can be used to assist pathologists in the early detection of lung and colon cancers. 

## 6. Discussion

In this study, CNN models with lower computational costs compared to previous studies are used to extract features from lung and colon cancer data. Experimental studies aim to propose a methodology with low computational cost but high accuracy. However, it should be noted that more costly and deeper CNN models can provide more powerful features. Moreover, the hyperparameter settings that are effective in the success of CNN models are determined manually for this study. Hyperparameter optimization methods can be used for a more successful CNN model. Different CNN models represent the same data with different features. Therefore, three different feature vectors are generated from ShuffleNet, MobileNet, and SqueezeNet CNN models to provide feature diversity and compare the different models. The size of the feature vectors is reduced by PCA, FHWT, and DWT. Size reduction can result in the removal of useful features while reducing the computational cost. However, this study does not take this into account, at the expense of developing a less costly method. In addition, the number of newly created features during feature reduction with PCA and FHWT affects system performance. In this study, the feature size is determined manually. Instead, systems that optimize the number of features can be developed. The strength of the proposed method is combining reduced features extracted from individual CNN models to increase feature diversity. In this way, at the classification stage, hybrid features produce more stable results than individual CNN features. However, extracting features from different CNN models is a temporal disadvantage. Instead, designing a single comprehensive deep model can provide more efficient results.

The most important requirement in deep learning-based diagnostic studies is the generalization ability and reliability of the network. In order to increase the number of training images, data augmentation methods are employed in this study. Although data augmentation is useful for deep learning studies, the new images produced are artificial and are a modified version of the original image. Few original images and many augmented images negatively affect the reliability of the artificial intelligence system. For this reason, using a large number of raw data, which are compiled by combining different datasets, provides results closer to reality.

The limitations and shortcomings of the proposed method discussed above will be the focus of our future work. The results obtained in our study showed that the proposed method can be used in histopathological image analysis for lung and cancer detection. Table 5 shows the methods and classification accuracy of previous studies using the same LC25000 dataset. In general, deep learning-based methods have provided successful results in previous studies. However, it is seen that the proposed method is comparable to previous studies in terms of accuracy.

## 7. Conclusions

Lung and colon cancer are the most common types of cancer worldwide. Early diagnosis is very effective in preventing deaths. In this study, a new methodology based on CNN and feature reduction methods is proposed for the early detection of lung and colon cancer cases. In the proposed methodology, the basic principle was to perform classification in an efficient and computationally low-cost manner. In this context, unlike previous studies, light CNN models were preferred. Instead of using a single model, features were extracted from the lung and colon images with the ShuffleNet, MobileNet, and SqueezeNet models to make comparisons, provide feature diversity, and benefit from hybrid features. PCA and FHWT and then DWT applied to these images reduced the computational cost, improved the variance between features, and offered a superior demonstration of the features used as input to machine learning classifiers. As a result of the application of individual methods to four different machine learning algorithms, high-accuracy diagnoses were achieved. In addition, with the classification of hybrid features obtained as a result of combining reduced features, more stable results were obtained compared to individual CNN models. The results from the proposed methodology showed that an application based on lightweight CNN models and feature transformation methods such as PCA, FHWT, and DWT provides superior accuracy, even though it contains fewer features and lower computational cost.

## Figures and Tables

**Table 1:** Classification accuracies (%) for several numbers of principal components after the PCA process conducted for the deep features of the three CNNs.

SqueezeNet				
Features	LDA	QDA	Linear SVM	ESD
PCA = 5	93.4	96.6	97.2	93.0
PCA = 10	96.0	96.7	97.7	94.5
PCA = 15	97.5	96.7	98.1	96.8
PCA = 20	97.5	96.4	98.1	96.7
PCA = 25	97.5	96.1	98.1	96.9
PCA = 30	97.6	96.0	98.1	96.9
**ShuffleNet**				
PCA = 5	92.8	96.8	97.4	91.7
PCA = 10	95.4	97.7	98.2	93.4
PCA = 15	96.4	97.8	98.5	94.8
PCA = 20	97.1	97.8	98.7	95.5
PCA = 25	97.1	97.8	98.7	95.5
PCA = 30	97.7	97.9	98.8	96.6
**MobileNet**				
PCA = 5	97.4	98.5	98.7	97.5
PCA = 10	98.1	98.6	99.0	97.9
PCA = 15	97.9	98.7	99.1	97.8
PCA = 20	98.3	98.8	99.1	98.1
PCA = 25	98.3	98.9	99.2	98.1
PCA = 30	98.4	98.9	99.2	98.3

**Table 2:** Performance measures attained using the combined PCA coefficients of the three CNNs.

Model	Sensitivity	Specificity	Precision	F1-Score	MCC
**LDA**	0.991	0.998	0.991	0.991	0.988
**QDA**	0.991	0.998	0.991	0.991	0.989
**Linear SVM**	0.996	0.999	0.996	0.996	0.994
**ESD**	0.989	0.997	0.990	0.989	0.987

**Table 3:** Classification accuracies (%) for different feature sets after the process conducted for the deep features of the three CNNs.

SqueezeNet				
Features	LDA	QDA	Linear SVM	ESD
50	97.7	95.3	98.0	97.6
40	97.8	96.5	98.1	97.6
30	97.8	96.9	98.1	97.6
20	97.8	97.5	98.2	97.6
10	97.8	97.7	98.2	97.6
**ShuffleNet**				
500	98.4	93.3	99.0	98.3
400	98.2	92.9	99.0	98.1
300	97.8	92.4	98.8	97.8
200	97.4	91.3	98.6	97.2
100	94.4	89.2	97.3	94.1
**MobileNet**				
500	98.7	97.1	99.2	98.7
400	98.5	97.0	99.2	98.5
300	98.4	96.8	99.1	98.3
200	97.8	96.2	98.8	97.8
100	96.8	94.3	98.1	96.7

**Table 4:** Performance measures attained using the combined FHWT coefficients of the three CNNs via the DWT method.

Model	Sensitivity	Specificity	Precision	F1-Score	MCC
**LDA**	0.993	0.998	0.993	0.993	0.991
**QDA**	0.993	0.998	0.993	0.993	0.991
**Linear SVM**	0.996	0.999	0.996	0.996	0.995
**ESD**	0.992	0.997	0.992	0.992	0.989

**Table 5:** Comparative analysis with previous works.

Authors	Method	Accuracy (%)
Masud, Sikder, Nahid, Bairagi, and AlZain [9]	CNN + 2D Fourier transform and 2D wavelet transform	96.33
Hasan, Ali, Rahman, and Islam [57]	CNN + PCA	99.80
Kumar, Sharma, Singh, Madan, and Mehandia [8]	DenseNet121 + Random Forest	98.60
Talukder, Islam, Uddin, Akhter, Hasan, and Moni [53]	Deep feature extraction + Ensemble learning	99.05
Bukhari, Syed, Bokhari, Hussain, Armaghan, and Shah [58]	ResNet50	93.13
Mangal, Chaurasia, and Khajanchi [54]	CNN	97.00
Hatuwal and Thapa [55]	CNN	97.20
Ali and Ali [56]	Capsule network model	99.58
Proposed method	PCA + CNN + SVM	99.5
	FHWT + CNN + SVM	99.6

## Data Availability

The data utilized in this work can be found at https://www.kaggle.com/datasets/andrewmvd/lung-and-colon-cancer-histopathological-images (accessed on 5 September 2022).
